# Complement Component 3: an assessment of association with AMD and analysis of gene-gene and gene-environment interactions in a Northern Irish cohort

**Published:** 2010-02-10

**Authors:** Gareth J. McKay, Shilpa Dasari, Christopher C. Patterson, Usha Chakravarthy, Giuliana Silvestri

**Affiliations:** 1 Centre for Vision and Vascular Sciences, Queen's University of Belfast, Belfast, Northern Ireland, UK; 2 Centre for Public Health, Queen's University of Belfast, Belfast, Northern Ireland, UK

## Abstract

**Purpose:**

A non-synonymous single nucleotide polymorphism (SNP) in complement component 3 has been shown to increase the risk of age-related macular degeneration (AMD). We assess its effect on AMD risk in a Northern Irish sample, test for gene–gene and gene–environment interaction, and review a risk prediction model.

**Methods:**

SNP rs2230199 was genotyped in 1,358 samples, which comprised 437 cases, 436 no-disease controls, and 485 participants randomly sampled from the Northern Ireland population. Allele frequencies were assessed in cases and controls. Logistic regression analysis was used to assess interaction and develop a risk prediction model.

**Results:**

We report a minor allele frequency of 0.248 for rs2230199 in the population (n=485), 0.296 in cases (n=437), and 0.221 in controls (n=436; odds ratio [OR]=1.48; confidence interval [CI]: 1.19–1.85; p=0.0003). The significant association is retained following multivariate analysis with adjustment for age, smoking status, Complement Factor H (*CFH*), Age-Related Maculopathy Susceptibility 2 (*ARMS2)*, Complement Component 2 (*CC2*), and Complement Factor B (*CFB*; OR=1.45; CI: 1.10–1.91; p=0.009). No evidence to support an interaction between any of the covariates within the regression model was found. The area under the receiver operator characteristic curve calculated for the fully adjusted model, including all variables, was 0.86 for late AMD.

**Conclusions:**

Our study confirmed the association between Complement Component 3 (*C3*) and late-stage AMD. There was no evidence for an interaction with environmental exposures, nor did we find data to support a gene–gene effect.

## Introduction

Age-related macular degeneration (AMD, MIM 603075) is a degenerative retinal disease that causes progressive impairment of the central vision and is the leading cause of irreversible severe vision loss in Caucasians over the age of 50 [1]. AMD begins with drusen formation and, in some cases, degenerative changes at the level of the retinal pigment epithelium (RPE), often referred to as early AMD, that primarily affects the macula and can progress to the visually disabling late phenotypes of geographic atrophy (GA) and/or neovascular AMD. Susceptibility to AMD is complex, involving genetic, lifestyle, and environmental factors, although the specifics of the etiology remain, in part, unresolved. Advances in our understanding of the genetic basis of the disease etiology have identified risk and protective haplotypes in several genes associated with the complement pathway and chronic inflammation, such as Complement Factor H (*CFH*) [2-6], Complement Component 2 (*CC2*)/ Complement Factor B (*CFB*) [7,8], and Complement Component 3 (*C3*) [9-11]. A dysfunctional complement pathway has been proposed to accentuate retinal cell damage with increased formation of drusen deposits, atrophy, and cell degeneration [12] and progression to choroidal neovascularization [5]. A non-synonymous single nucleotide polymorphism (SNP), rs2230199 (R102G), which causes an amino acid change from arginine to glycine in *C3*, has been most commonly associated with late-stage AMD in other studies, primarily due to the functional nature of this variant, which differentiates between the electrophoretic allotypes of C3. As high linkage disequilibrium (LD) exists between rs2230199 and rs1047286 (P314L), the latter cannot be excluded as a functional variant on the basis of a genetic signal [11].

Beyond the complement pathway, the Age Related Maculopathy Susceptibility 2* (ARMS2)* locus at chromosome 10q26 has been implicated as the other major genetic contributor to the AMD disease process [13-16]. Although discussion continues regarding the source of the genetic effect observed at this locus due to high LD within the region, data have been reported supporting mitochondrial involvement through interaction with the translocase of outer mitochondrial membrane proteins and co-localization to the mitochondrial-rich ellipsoid region of the photoreceptors [17,18]. Mitochondrial dysfunction and resultant increased oxidative stress have been implicated as a component of AMD disease etiology, with reports of increased mitochondrial damage in the neural retina and RPE with aging, particularly in AMD. Variation within the mitochondrial genome at NADH ubiquinone oxidoreductase chain 2 (*MTND2* A4917G) has been reported to confer increased risk associated with AMD [19,20] and as such would provide an excellent biologic candidate of functional significance, although subsequent replication and validation in independent cohorts is necessary. Here, we assess the effect of the *C3* functional variant rs2230199 independent of *CFH*, *ARMS2*, *CC2*, *CFB*, age, and smoking status in a Northern Irish cohort; we check for potential gene–gene or gene–environment interactions, and we use the area under the receiver operator characteristic (ROC) curve to quantify the predictive ability of a logistic regression model, incorporating both genetic and environmental risk factors.

## Methods

### Study participants

Patients with end-stage AMD (n=437; average age=77.6 years; 39.1% male), either GA or neovascular in at least one eye were recruited opportunistically between June 2002 and September 2006 from ophthalmology clinics in the Royal Victoria Hospital, Belfast, UK, which is the regional referral center for Northern Ireland. DNA was available from an age-matched control group (n=436; average age=74.9 years; 38.5% male) recruited through random sampling of older adults (aged 65 years and above) resident in Northern Ireland and who consented to stereoscopic digital fundus photography and DNA analysis to ascertain disease status. Fundi of control participants were either free of any drusen or had fewer than five hard drusen of diameter less than 63 µm and absence of focal pigmentary irregularities, such as hyperpigmentation or hypopigmentation, with image grading undertaken by trained reading center staff using the Wisconsin Age-related Maculopathy Grading System [21]. All participants were from Northern Ireland and described themselves as of European descent. An additional control sample (n=485) obtained through random sampling of the population from across Northern Ireland in 2006 was used to assess allele frequencies only but had not been assessed for AMD disease status.

### Clinical assessment of cases

Participants recruited from ophthalmology clinics had a confirmed diagnosis of late AMD made following clinical examination and fluorescein angiography [21]. In 32 participants, the diagnosis was advanced atrophic AMD, i.e., geographic atrophy, and 405 participants were diagnosed with exudative AMD. These late-stage manifestations were present in at least one eye of each participant. Cases with macular pathology due to other primary causes that mimic neovascular AMD, such as myopic degeneration, adult vitelliform, central serous retinopathy, diabetic retinopathy, or idiopathic macular telangiectasia, were excluded. Those with lens opacities sufficient to obscure retinal detail were also excluded. A history of cigarette smoking was ascertained at the time of examination, and the questionnaire used had been previously validated in the Whitehall study [22], with participants classified as never smokers, ex-smokers or current smokers.

Informed written consent was obtained from all subjects involved. The study was approved by the Research Ethics Committee of the Queen's University of Belfast and the Office for Research Ethics Committee, Lisburn, Northern Ireland and conformed to the tenets of the Declaration of Helsinki.

### Genotyping of AMD susceptibility loci

DNA was extracted from peripheral blood leukocytes or frozen buffy coat samples using standard protocols in accordance with the manufacturers’ instructions (Promega Wizard® Genomic DNA Extraction Kit, Southampton, UK). Genotyping was undertaken by multiplex PCR and primer extension methodology using standard manufacturer’s protocols (ABI Snapshot, ABI, Warrington, UK) using custom-designed primer and probe sequences (Table 1) to ascertain the genetic data for rs1061170, rs800292, rs419137, rs6677604, rs2284664, and rs3753396 in *CFH*; rs10490924 in *ARMS2*; rs9332739 in *CC2*; rs641153 in *CFB;* and rs2230199 in *C3*.

**Table 1:** Snapshot PCR primer and probe sequences

**SNP**	**Gene**	**Primer and probe sequence (5′-3′)**
rs10490924	*ARMS2*	F: TTATGTCCCTGTACCCTACATGC
		R: AGGAGAGAAGAAGGCTGGTAAGC
		Probe: TCACACTCCATGATCCCAGCT
rs419137	*CFH*	F: CAGCTATACCACTGATGTAGAGG
		R: CCTACTTACTACTCTCCCATAGG
		Probe: GACTGACTGACCACCAACCCTGCAGCACATT
rs6677604	*CFH*	F: ACCAGAGCAGATACAGCAAAAGG
		R: AAGCACAATACCTCCACAGTAGC
		Probe: ACCCCCCCCAGTTGCCCTGAGAAAATGCGAG
rs2284664	*CFH*	F: GTCATCCATCAAGTGCTACAACC
		R: CAGTGGAAGTATGTGCCCTAAGC
		Probe: CATCATCATCATAGAAAAATACCAGTCTCCATAGATC
rs3753396	*CFH*	F: CACCTCCTGAACTCCTCAATGG
		R: ACTGGTAAAGTTGTCCACTCTCC
		Probe: AGACAGACAGACAGACATTTGTCCACTCTCCATCAACACA
rs1061170	*CFH*	F: TTTATCATTGTTATGGTCCTTAGG
		R: AGTGTACTTACTGACACGGATGC
		Probe: GGACGGACGGACGGACGGACTCCCTGTACAAACTTTCTTCCAT
rs800292	*CFH*	F: GGATTAAGAGCAACCCATTCTCC
		R: CTGACCAAACATATCCAGAAGGC
		Probe: AAAAAAAAAAACCCCCCCCCCCCCCTGGATATAGATCTCTTGGAAAT
rs9332739	*CC2*	F: GCGTTGCCATTATCACCTTTGC
		R: CCTCTCTCATCACCATCACGTGA
		Probe: CCCAAAAACATTTTCCAGGCTGCTGATCAC
rs641153	*CFB*	F: AGCAAGCCAGGACACACCATCC
		R: GGAGCCGCCTTTGATCTCTACC
		Probe: CCCCCCCCAAAAAACAGAGAGCAGGATCCCTGGGGC
rs2230199	*C3*	F: CAGGGAGTTCAAGTCAGAAAAGG
		R: TCTTGTCTGTCTGTCTGGATGAAGAGG
		Probe: CCCCCCCCCCCCCCCCCCCCCCCCCCCGGCCTGCACGGTCACGAACTTGTTGC

### Statistical analysis

All SNPs were verified, validated, and assessed for Hardy–Weinberg equilibrium (HWE) separately in cases and controls, using a χ^2^ goodness-of-fit test. Allele frequencies for the SNPs being tested were compared in cases and controls by the Pearson's χ^2^ test of association. The assessment of associations of genetic markers with AMD risk and interactions between genetic markers and between genetic markers and smoking were obtained using likelihood ratio χ^2^ tests in a logistic regression model. Age was included in the model as a continuous variable. Tests for trend for the number of risk alleles for each genetic variant (0, 1, or 2) were calculated. Initially only rs2230199 was fitted in the model and additional covariates were then added to adjust for their possible confounding effect. *CFH* haplotypes were entered into the logistic regression model as a count (0, 1, or 2) depending on the number of copies of the haplotype an individual carried. The lowest risk haplotype was omitted from the model to avoid collinearity problems so that odds ratios (ORs) were measured relative to homozygote carriers of this haplotype. For our final risk analysis, the number of copies carried for rs10490924, rs9332739, and rs641153 were coded 0, 1, or 2. Tests for interactions between each of the genotypes and between genotypes and smoking were used to check for effect modification. The fitted logistic model was used to order individuals in terms of their predicted AMD risk dependent on their rs2230199, *CFH*, rs10490924, rs9332739, and rs641153 genotypes and their smoking status.

A value for the area under the ROC curve, also known as the C statistic, was evaluated by using only phenotypes from either end of the severity spectrum, i.e., those with symptoms of end-stage disease only and those with no features of early AMD (namely pigmentary irregularities or soft drusen deposits less than 63 μm). A method to compare the area under two ROC curves derived from paired measurements was used to assess the value of adding extra variables to the prediction equation [23].

## Results

### Univariate analysis

Genomic SNP rs2230199 was assessed by a χ^2^ test and was found to be in HWE in both cases and controls. ORs, confidence intervals (CI), and associated p values are shown in Table 2. A minor allele frequency of 0.248 was recorded for rs2230199 in the general population (n=485), and a frequency of 0.296 in cases (n=437) and 0.221 in controls (n=436; OR=1.48; CI: 1.19–1.85; p=0.0003), similar to reports from previous studies [9-11].

**Table 2:** Genotype and allele frequencies, odds ratios, confidence intervals (95%) and p-values for rs2230199 in 437 cases of AMD and 436 controls.

**Genotype**	**Cases n (%)**	**Controls n (%)**	**Odds ratio (95%CI)**	**p value**
CC	220 (50.3)	270 (61.9)	1.00 (reference)	
CG	175 (40.0)	139 (31.9)	1.55 (1.15–2.08)	0.003
GG	42 (9.7)	27 (6.2)	1.91 (1.11–3.30)	0.013
C allele	615 (70.4)	679 (77.9)	1.00 (reference)	
G allele	259 (29.6)	193 (22.1)	1.48 (1.19–1.85)	0.0003

### Multivariate analysis

The significant effect associated with rs2230199 was maintained after adjustment for the effects of age, cigarette smoking status, and carriage of the adverse alleles of *CFH*, *ARMS2,* and *CC2/CFB* (Table 3: OR=1.45; CI=1.09–1.91; p=0.009).

**Table 3:** Estimates of AMD risk from a logistic regression model with terms for smoking, *CFH* haplotype [6], genotype at *ARMS2* A69S (rs10490924) and the risk associated with the *CC2/CFB* region [8].

**Regression term**	**OR**	**95% C.I.**		**Significance**
Ex-smoker versus never smoker	2.34	1.59	3.5	<0.001
Current smoker versus never smoker	5.04	2.96	8.58	<0.001
Number of *CFH* haplotype 1	3.66	2.24	5.98	<0.001
Number of *CFH* haplotype 2	4.5	2.85	7.09	<0.001
Number of *CFH* haplotype 3	2.95	1.8	4.81	<0.001
Number of *CFH* haplotype 4	1.68	1.01	2.81	0.047
*ARMS2* A69S heterozygous	3.64	2.47	5.36	<0.001
*ARMS2* A69S homozygous	25.3	13.6	47.2	<0.001
rs641153 (*CFB*)	0.3	0.18	0.5	<0.001
rs9332739 (*CC2*)	0.55	0.3	1.01	0.056
rs2230199 (*C3*)	1.45	1.1	1.91	0.009
Age/year	1.1	1.08	1.13	<0.001

Risks associated with smoking are relative to never smokers. *CFH* haplotypes (1–4; haplotypes 1 and 2 increase risk and are in complete LD with the C risk allele at Y402H while 3 and 4 are non-risk and in complete LD with the T allele at Y402H) are measured against haplotype 5 (the most protective haplotype, which is in complete LD with the deletion at *CFHR-3*) [6]. *ARMS2* is measured on the heterozygosity or homozygosity of the A69S risk allele (rs10490924). rs641153 (*CFB*), rs9332739 (*CC2*) and rs2230199 (*C3*) were coded as 0, 1 or 2 depending on the number of copies of the minor allele present.

### Receiver operator characteristic curves

**Figure 1:**
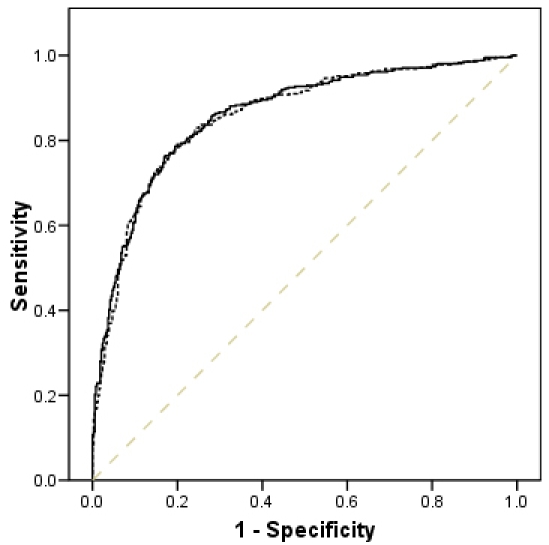
Sensitivities and specificities for a variety of risk score cutoffs and area under the receiver operator characteristic (ROC) curve for prediction of age-related macular degeneration. The area under the ROC curve was 0.860 (95% confidence interval [CI]: 0.834–0.885; p<0.001) for cases versus controls with the covariates *CFH, ARMS2, CC2, CFB*, *C3*, smoking status, and age fitted in the regression model (solid line). The area under the ROC curve showed a non-significant reduction (dotted line; p=0.22) to 0.857 (95% CI: 0.831, 0.883; p<0.001) upon removal of *C3*. The dashed line represents a reference area of 0.50.

An ROC curve for cases versus controls with the covariates *CFH, ARMS2, CC2, CFB*, *C3*, smoking status, and age fitted in the regression model is presented in Figure 1. The area under the ROC curve that was generated from the model using all covariates was 0.860 (95% CI: 0.834, 0.885; p<0.001), which showed a non-significant (p=0.22) reduction to 0.857 (95% CI: 0.831, 0.883; p<0.001) upon removal of *C3*.

### Potential gene–gene and gene–environment interactions

Potential gene–gene and gene–environment interactions were assessed using logistic regression for all the combinations of *C3* and the other parameters recorded within the full model, such as *CFH, ARMS2, CC2, CFB*, smoking status, and age. There was no evidence to support any interaction between the parameters measured.

## Discussion

The polymorphic variation measured for the non-synonymous SNP rs2230199 in *C3* shows significant association with late AMD in the Northern Irish population and supports the previously noted dose effect with an increased risk of 1.55 for heterozygotes (CI: 1.15–2.08; p=0.003) and 1.91 for homozygotes (CI: 1.11–3.30; p=0.013) [9-11]. The OR per allele copy decreases slightly from the univariate value calculated following adjustment through regression analysis for the following covariates: *CFH, ARMS2, CC2, CFB*, age, and smoking (OR=1.45; CI: 1.10–1.91; p=0.009). The minor allele frequency of rs2230199 ascertained from the general population sample (n=485) approximates at 0.25.

Considerable LD exists across this region, with the majority of investigations reporting the association observed at this locus on the basis of the genotype at rs2230199 [9-11]. With evidence supporting a biologic functional effect through the formation of two common allotypes of C3 (C3F [fast] and C3S [slow]), primarily due to variation in the electrophoretic mobility, the rationale for targeting this SNP for association studies is strong [24]. However, Park et al. [11] presented data supporting extensive LD within the region and suggested that until functional data are produced supporting rs2230199 as the causal variant in AMD disease etiology, it is important to consider the potential role of the non-synonymous SNP rs1047286 (P314L), which is in high LD with rs2230199, or indeed the potential role of any additional and as yet undetermined variants that may also be in complete LD.

Inclusion of rs2230199 as a variable has helped refine our AMD disease prediction model, providing an area under the ROC curve of 0.860 (Figure 1). Seddon et al. [25] reported an ROC curve of 0.831 in a longitudinal study of progression to advanced AMD, using a model incorporating information on the genetic loci rs1061170 and rs1410996 in *CFH*; rs19490924 in *ARMS2*; rs9332739 in *CC2*; rs641153 in *CFB;* and rs2230199 in *C3* in addition to the response to therapeutic supplementation. Our ROC area was comparable to that of Seddon et al. [25], despite the cross-sectional nature of our study. The similarity of the values determined from this study to that reported by Seddon et al. [25] may be due to the homogeneity of our sample in terms of the late-stage disease phenotype, which could have contributed to an improved ROC area. Also, we used an informative representation of the *CFH* locus identifying five haplotypes [6] as opposed to the binary format of the Y402H locus, which is used in most investigations. Although Seddon et al. [25] used two SNPs, offering improved power over Y402H alone, we contend that there is merit in using the haplotypic structure of this locus, which improved the ROC statistic [6]. This haplotype is based upon several SNPs from this region, which enable the differentiation of five haplotypes, including the deletion at the *CFHR1–3* locus. It was interesting to note that addition of rs2230199 within our regression model did not significantly improve the area under the ROC curve, with only a moderate increase from 0.857 to 0.860. Evaluation of risk prediction models requires model performance measures, such as the popular ROC metric [23]. Its probabilistic interpretation is based upon the ability of the models’ predicted probabilities to correctly discriminate between randomly selected diseased and nondiseased subjects. However, it is known that the area under the ROC curve often shows only small and nonsignificant improvements, even for a predictor variable that makes a significant independent contribution in a logistic regression analysis. More recently, alternative methods for evaluating the usefulness of a new marker have been proposed, such as event-specific reclassification tables and integrated (average) sensitivity, although these methodologies are more useful in cohort than case-control studies [26].

In conclusion, the *C3* locus has a small but significant role in the etiology of the disease process of AMD in comparison to the other established genetic risk factors. Smoking and the genetic risk factors were found to exert an independent effect in this cohort study. While this conclusion supports the findings of other independent investigations, reports have been published supporting gene–gene or gene–environment interactions in AMD. Perhaps it would be appropriate to exercise caution in defining interaction on the basis of single studies but implementation of replication in independent data sets in a manner similar to that required for purely genetic association studies would provide a more robust measure.

Our findings add to the literature on calculating AMD risk and could, in time, facilitate earlier practical intervention for those at risk, with either lifestyle modification strategies or preclinical therapeutic intervention when these become available. These models will also enable us to identify predicted high-risk individuals who are aged and have not progressed to a severe disease phenotype or, conversely, individuals with low predicted risk and disease, for inclusion within future studies for elucidating novel risk factors that contribute to the AMD disease process.
